# Antibiotic prescribing and dispensing for acute respiratory infections in children: effectiveness of a multi-faceted intervention for health-care providers in Vietnam

**DOI:** 10.1080/16549716.2017.1327638

**Published:** 2017-06-07

**Authors:** Nguyen Quynh Hoa, Pham Thi Lan, Ho D. Phuc, Nguyen Thi Kim Chuc, Cecilia Stalsby Lundborg

**Affiliations:** ^a^ Department of Pharmacy, Vietnam National Cancer Hospital, Hanoi, Vietnam; ^b^ Department of Dermatology, Hanoi Medical University, Hanoi, Vietnam; ^c^ Department of Probability and Statistics, Institute of Mathematics, VAST, Hanoi, Vietnam; ^d^ Division of Global Health (IHCAR), Department of Public Health Sciences, Karolinska Institutet, Stockholm, Sweden

**Keywords:** Antibiotics, acute respiratory tract infections, health-care provider, intervention, Vietnam

## Abstract

**Background**: Appropriate antibiotic use is vital to effectively contain antibiotic resistance and improve global health. Acute respiratory infections (ARIs) remain the leading cause of disease and death in children under five in low-income countries.

**Objective**: To evaluate a multi-faceted intervention targeting health-care-providers’ (HCPs) knowledge, practical competences and practices regarding antibiotic use for ARIs.

**Methods**: A multi-faceted educational intervention with a two-armed randomised controlled design targeting HCPs treating ARIs in children was conducted in Bavi district, a rural district in Northern Vietnam in 2010–2011. Thirty-two communes of the district were randomized into two arms, with 144 HCPs in the intervention arm and 160 in the control arm. The intervention, conducted over seven months, comprised: (i) education regarding appropriate-antibiotic use, (ii) case scenario discussion and (iii) poster distribution. Questionnaires to assess knowledge and dispensing/prescribing forms to assess practice were completed before-and after interventions. The main outcome measures were differences in improvement in knowledge and practice in the intervention and control group, respectively.

**Results**: Knowledge improved in the intervention group for ARI aetiology by 28% (Δ_Decrement control arm_ 10%), antibiotic use for mild ARIs by 15% (Δ_Decrement control arm_ 13%) and for severe ARIs by 14% (Δ_Improvement control arm_ 29%). Practical competence for a mild ARI case scenario improved in the intervention and control groups by 20% and 11%, respectively. Total knowledge score increased statistically in the intervention group (Δ_mean improvement_ 1.17); less so in the control group (Δ_mean improvement_ 0.48). Practice regarding antibiotics for mild ARIs improved by 28% in the intervention group (Δ_Decrement control arm_ 3%).

**Conclusions**: The intervention significantly improved HCPs’ knowledge of ARIs and practice of antibiotic use in treatment of ARIs. We suggest mixed method assessment and long-term follow-up of these interventions to enable better appreciation of the effects and effect sizes of our interventions.

## Background

Antibiotics are life-saving drugs with rapidly declining effects worldwide due to increased antibiotic resistance, a natural consequence of all antibiotic use [1–3]. Resistance is considered a threat to global public health, and containment of resistance is thus a ‘global public good’. A correlation has been shown at country level as well as at the individual level between antibiotic use and antibacterial resistance [4]. The problem of irrational antibiotic use has been reported in many countries, including Vietnam which has noted a rapid increase of antibiotic resistance in recent years [5–7]. Worldwide, misconceptions and subsequent practice of dispensing/prescribing antibiotics among health-care providers (HCPs) may be important factors behind the increasing antibiotic resistance [8,9].

In Vietnam, acute respiratory infections (ARIs) remain the leading cause of morbidity and mortality in children [10]. A high prevalence of antibiotic resistance and multi drug resistance of *Streptococcus pneumoniae*, the major cause of bacterial ARIs, has been seen in Vietnam [3,11]. The reality of drug resistance is countered in practice by changing empirical therapy through replacing assumed ineffective first-line antibiotics with second- or third-line antibiotics. These drugs are more costly, occasionally harmful, often unavailable, and frequently may not be necessary [12].

Irrational antibiotic use for children has been reported in Vietnam among prescribers, dispensers and consumers [13,14] although Integrated Management of Childhood Illness (IMCI) had been formulated internationally and adapted in national guidelines [15–17]. Antibiotics are frequently dispensed or prescribed in drug stores, commune health stations or private clinics, often without an appropriate indication [18–20]. It has been reported that most HCPs prescribe or dispense antibiotics for common colds and only 19% had knowledge compliant with the recommended guidelines. A serious lack of knowledge among HCPs regarding the pathogeneses of ARIs and the ability to recognize the signs for pneumonia has been noted [18–20].

A wide range of factors might influence the very high level of antibiotic prescribing and dispensing for treatment of mild ARIs by HCPs. Antibiotic prescribing and dispensing has been high due to lack of knowledge, due to pressure to meet patients’ antibiotic expectations and due to misconceptions that antibiotics should be used to prevent secondary bacterial infections and complications of disease [13,18]. In Vietnam, multi-faceted interventions have previously been conducted in private pharmacies and have improved antibiotic dispensing patterns for childhood ARIs [21]. However, there has been a lack of randomized controlled trials evaluating interventions to improve the knowledge and practices of HCPs regarding classification, treatment and prescribing/dispensing antibiotics for ARIs. The aim of this study was therefore to evaluate the effects of a multi-faceted intervention on the knowledge and practices of HCPs regarding antibiotic use for ARIs among children in rural Vietnam and to examine which determinants may possibly impact the intervention’s effectiveness.

## Methods

We conducted a two-armed cluster randomised controlled trial to evaluate the effects of a multi-faceted intervention on antibiotics prescribing/dispensing for ARIs.

### Study setting and participants

This study was a part of a randomised controlled educational intervention conducted in Bavi district, Hanoi, Vietnam in 2010 and 2011. Bavi district is a rural district, 60 km west of Hanoi City. It covers 410 km^2^ and has a population of approximately 235,000 people within 32 communes. The basic public health care system includes a district hospital with 150 beds, 3 regional polyclinics and 32 commune health stations (CHSs), village health workers, private facilities and pharmacies/drugstores.

The study participants included all medical personnel (medical doctors, assistant medical doctors, nurses and midwives) and pharmacy personnel (pharmacists and drugsellers) working in public and private facilities in the district.

### Design and intervention

**Figure 1.**
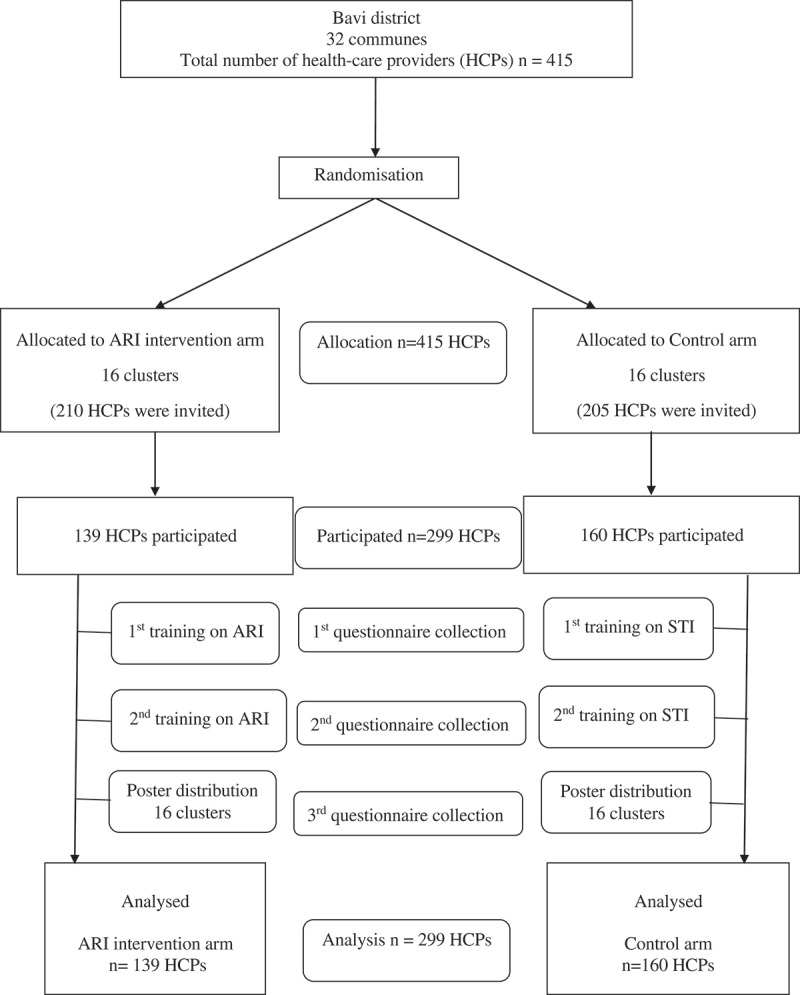
Flow chart of the study.

A randomised controlled trial, with randomisation at commune level, was conducted to examine the effect of a multi-faceted educational intervention on knowledge and reported practice regarding sexually transmitted infections (STI) and ARIs among HCPs. The 32 communes of Bavi district were randomized by computer into two arms (16 communes per arm), an STI intervention arm and an ARI intervention arm. All HCPs working in the communes were invited to participate. Each arm got active intervention for one topic and functioned as the control arm for the other topic (Figure 1) to control for the non-specific intervention effects (Hawthorne or attention effects). The results of the interventions on STI were presented in another paper [22]. Changes in the HCPs’ knowledge after the intervention were assessed through a self-completed questionnaire, and changes in practices were assessed through prescribing/selling forms.

In the ARI intervention arm, three activities were performed sequentially from September 2010 to March 2011, including: (i) six training sessions on ARI management and appropriate use of antibiotics based on IMCI guidelines (one day each session); (ii) six training sessions on ARI case scenario management; and (iii) poster distribution. In each training session (one day each session), 25–30 HCPs participated and were divided into 4 or 6 small groups (approximately 5–7 HCPs per group) to discuss different issues related to ARI. Afterwards, each small group presented their group work results, which were then discussed with the entire group. Finally, the trainers gave correct information and a lecture on the topics discussed. For the first training, the issues discussed focused on ARI knowledge and appropriate antibiotic use; classification and treatment of specific symptoms of ARIs; and local antibiotic resistance patterns to the major respiratory pathogens. Guidelines and algorithms for ARIs in children were developed and distributed to assist HCPs in deciding when to use antibiotics.

The second training included full-day discussion of ARI management and communication skills of HCPs with child caregivers. The case scenario was based on IMCI guidelines and focused on distinguishing mild and severe ARIs and when to use antibiotics and when not to use antibiotics [15,16,23]. The case scenario role plays consisted of participants interacting as (i) HCPs and (ii) patients. In the case scenario of mild ARIs, delayed antibiotics were discussed as a means of demonstrating to patients that antibiotics are not always necessary, while providing adequate control of symptoms to get high levels of patient satisfaction.

**Figure 2.**
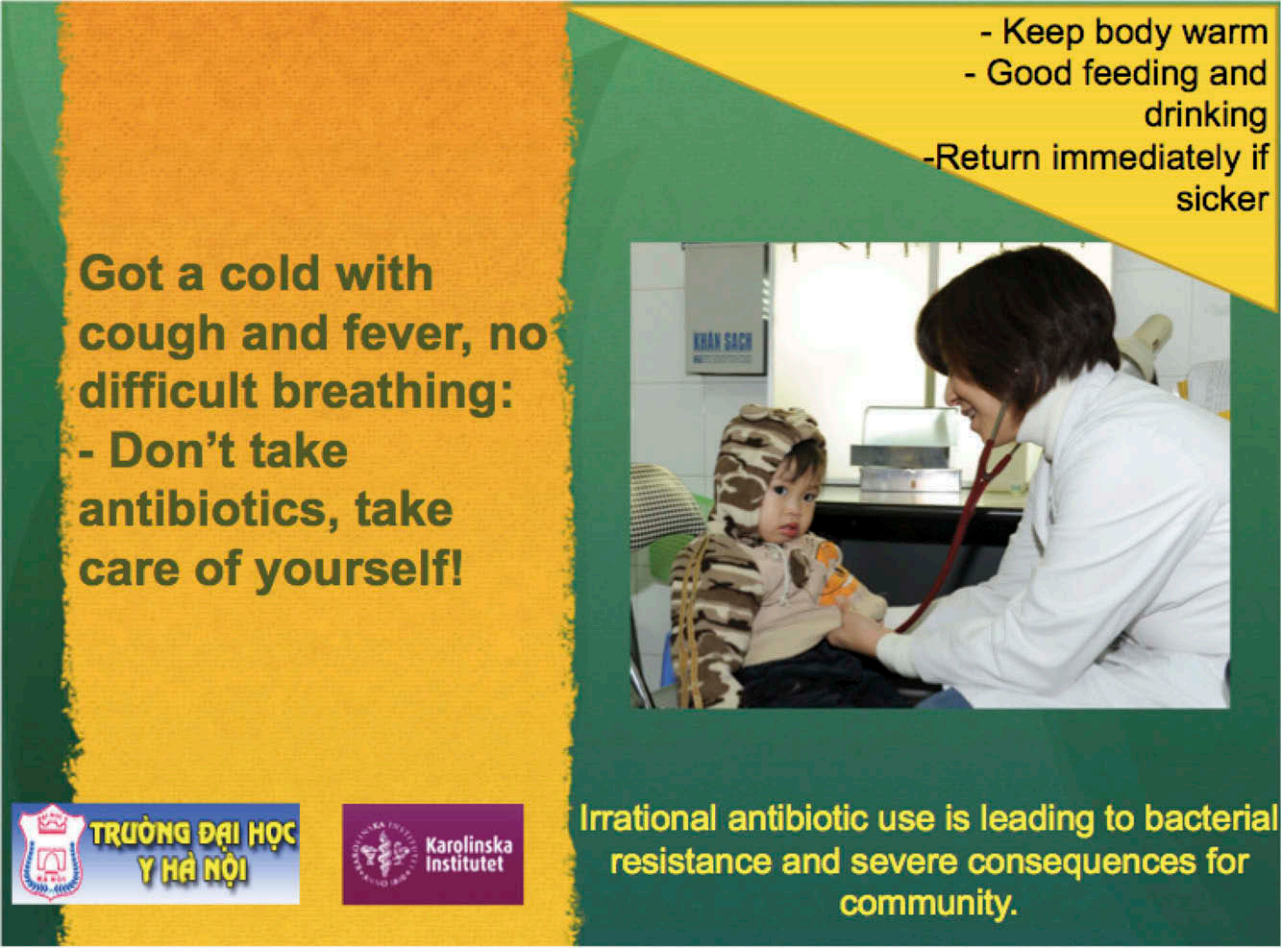
The posters for the health care facilities in Bavi district.

The third intervention consisted of poster distribution in the health care facilities at which the participants in the ARI arm worked. The posters were based on IMCI guidelines, including messages focusing on taking care of children with mild ARIs [16,23] (Figure 2).

The trainers of the ARI intervention arm were medical doctors and lecturers from Hanoi Medical University who specialized in paediatrics, and in antibiotic use with an interest in ARIs. The interventions for the STI arm consisted of three educational interventions at the same time periods as the ARI interventions. The educational STI sessions were conducted by individuals with equivalent expertise in STIs.

### Questionnaire

The questionnaire included an STI part and an ARI part. For the ARI intervention arm, the ARI part was presented just after questions on HCPs’ characteristics. Similarly, for the STI arm, an alternative version of the questionnaire was constructed with the STI section first, followed by the ARI part. The ARI section of the questionnaire was created contextually through previous studies in the same environment [18] and also using World Health Organization (WHO) IMCI guidelines [16,23]. It included two parts: (i) knowledge about etiology and antibiotic use for ARI symptoms among children under five; (ii) clinical scenarios examining practical competence related to the management of children under five with ARIs.

### Data collection

Triplicate assessments were held at baseline and two months after each intervention (intermediate-term effect). The questionnaire was pre-tested outside the study setting several times before being given to 144 HCPs in the intervention arm and 160 HCPs in the control arm: (i) prior to the first intervention, (ii) between the first and second interventions and (iii) after the last intervention. The research team asked participating HCPs to individually complete the questionnaire without any discussion or assistance, and then collected it back on the same day.

Prescription patterns were assessed by the distribution of 35 prescribing/selling forms per HCP within one month pre- and post- interventions. Thirty HCPs were randomly selected to complete the forms. Subsequently, prescribing forms, i.e. by health commune station staff and private clinic practitioners, and dispensing patterns, i.e. by drug sellers, were completed twice: (i) before the initial intervention and (ii) after all interventions.

### Analysis and outcomes

ACCESS software was used for data entry, and SPSS version 20.0 (IBM Corporation, San Francisco, USA) and STATA version 11 (Stata Corp LP, Texas, USA) for data analyses. Proportions, mean, median, minimum and maximum values were used for the descriptive analyses. Overall ARI knowledge and practice competence was evaluated by scoring, summing (maximum score eight) and calculating the mean scores. The outcome for practice of HCPs was calculated by the decrement proportion of antibiotic prescribing for treatment of mild ARIs.

The McNemar paired sample test was used to verify the proportion trend of correct answers before and after the intervention. Wilcoxon’s test was used for univariate comparisons of providers’ knowledge before and after the intervention within the intervention/control groups. The Mann–Whitney test was used to compare knowledge improvements between the ARI intervention and control arms. Two-level regression analysis was applied to determine which factors influenced the improvements. Each observation of an individual HCP can be seen as a unit in the first level, while each commune can be treated as a unit in the second level of the model.

Regression coefficients and *p*-values in the Wilcoxon and McNemar tests were adjusted for intra-cluster correlation (ICC) [24].

### Ethical considerations

The verbal consent that was obtained from all participants followed three steps. (1) The researcher (NQH) provided all the information including objectives, procedures, benefits etc. of the study to the HCPs. (2) The HCPs could ask questions and the researcher answered. The participants (HCPs) were informed about the interventions, that they could withdraw from the study at any time and that the data was treated anonymously. (3) After obtaining verbal agreement to participate in the research, the research team gave the self-completed questionnaire to the participants for filling out. Confidentiality was assured and the participants were again informed that they had the right to withdraw from the study at any time without needing to provide explanation. The Ethical Review Board of Hanoi Medical University (Vietnam) approved this study including the consent procedure (2010).

## Results

### Background characteristics of HCPs

In all, 284 of 415 targeted HCPs participated in the study (intervention: 130; control arm: 154). Reasons for non-participation (131 HCPs) included: an insufficiently up-to-date list of HCPs; incorrect contact addresses; and non-attendance or withdrawal by a total of 90 drug sellers and private providers. The characteristics of the participating HCPs are presented in Table 1.

**Table 1.** Socio-demographic characteristics of the participating health-care providers (HCPs) in the intervention and control arms.

	Absolute number and percentage of HCPs in each group	
	Intervention (n = 130)	Control (n = 154)
Characteristics	n (%)	n (%)
Age (years)		
≤ 29	25 (19)	34 (23)
30–39	32 (25)	42 (27)
40–49	39 (30)	39 (25)
50+	34 (26)	39 (25)
Sex		
Male	43 (33)	55 (36)
Female	87 (67)	99 (64)
Educational level*		
Basic^†^	29 (22)	14 (9)
Intermediate^††^	89 (69)	122 (79)
University^†††^	12 (9)	18 (12)
Practice type		
Public health facilities	74* (57)	108* (70)
Private clinics	33* (25)	17* (11)
Drug stores	23* (18)	29* (19)

Notes: *Significant difference using chi-square test (*p* < 0.05), comparing intervention and control arms.

^†^Basic drug seller, basic nurse, village health worker or no medical training.

^††^Assistant doctor, assistant pharmacist or middle nurse.

^†††^Medical doctor or pharmacist.

Most participants were working in public health facilities in both the intervention (57%) and control arms (70%). The second most common practice type was private health facilities (25%) in the intervention arm, and drug stores (19%) in the control arm. Most HCPs working at public facilities had an intermediate education (i.e. they were assistant pharmacists/doctors or middle nurses); meanwhile, a basic education (i.e. basic drug sellers, basic nurses, village health workers or no medical training) predominated at private facilities and drug stores.

### HCPs’ knowledge and practical competences

After all interventions, the percentage of correct answers for ARI knowledge questions increased in the intervention arm for all eight questions (Table 2). A greater improvement was observed in the intervention arm (with four of seven questions showing significant improvements), as compared with the control arm (with only one of seven questions showing significant improvement). Knowledge concerning aetiology of ARI improved significantly in the intervention arm but there was no significant change in the control arm. In contrast, no significant improvement was seen in distinguishing between pneumonia and a common cold. Knowledge increased significantly in the intervention arm for three questions (Q1,4,5) regarding antibiotic-requiring symptoms, compared with a significant improvement in only one of these questions (Q5) in the control arm. Correct answers regarding antibiotics for children with cough and fever but not fast breathing (Q6) improved in the intervention arm but showed a decrease in the control arm.

**Table 2.** Percentages of participants with correct answers and improvements for HCPs’ knowledge and practical competences regarding appropriate antibiotic use for ARI treatment.

Questions regarding HCP knowledge and practical competence	Percentage of participants with correct answers							
	ARI intervention group				Control group			
	Before	After	Difference	*p*-value^†^	Before	After	Difference	*p*-value^†^
Q1. Aetiology of ARI	21	49	28	0.000	31	21	–10	0.192
Q2. Distinguishing between pneumonia and a common cold	53	59	6	0.453	59	68	9	0.255
Q3. Symptoms don’t require antibiotics: cough and runny nose	75	85	10	0.172	70	74	4	0.505
Q4. Symptoms require antibiotics: cough, fever and fast breathing	75	89	14	0.015	83	87	4	0.509
Q5. Symptoms don’t require antibiotics: cough, no fever or chest contraction	75	89	14	0.043	47	76	29	0.000
Q6. Symptoms don’t require antibiotics: cough and fever	55	70	15	0.068	58	45	–13	0.123
Q7. Case scenario: probable diagnosis?	80	88	8	0.266	70	83	13	0.070
Q8 Case scenario: treatment for a child with cough and fever, not fast breathing?	37	57	20	0.023	32	43	11	0.059

Note: ^†^*p*-value in the McNemar test comparing the percentages of correct answers of HCPs before and after the interventions in each group, adjusted with respect to intra-cluster correlation (ICC).

Practical competence at case scenarios (Q8) improved significantly in the intervention arm but not in the control arm. The results from the questions on both knowledge and practical competence were in line with each other and indicated improved knowledge of HCPs in the intervention arm for the correct management of cough and fever, i.e. mild ARI.

Total scores were calculated by a sum of correct answers to the knowledge and practical competence questions (ranging between zero and eight). Univariate analysis (Wilcoxon) showed that the mean total knowledge score improved significantly in the intervention group in all subgroups, except in HCPs aged 40–49 years old and those with university education (Table 3). The mean total knowledge score improved significantly in the intervention arm, with the mean scores and the mean improvement in the intervention arm (4.7–5.87, Δ_mean improvement_ 1.17) found to be higher than those in the control arm (4.49–4.97, Δ_mean improvement_ 0.48).

**Table 3.** Mean of total knowledge scores for HCPs regarding appropriate antibiotic use for ARI treatment in children under five, divided according to characteristics.

Characteristics	Mean of total knowledge score (range: 0–8)								Improvement difference between intervention and control groups	*p*-value ^††^
	ARI intervention				Control					
	Before	After	Improvement	*p*-value^†^	Before	After	Improvement	*p*-value^†^		
Age (years)										
≤ 29	4.68	6.16	1.48	0.001	4.24	4.56	0.32	0.369	1.16	0.078
30–39	4.94	6.19	1.25	0.019	4.71	5.33	0.62	0.188	0.63	0.309
40–49	4.64	5.38	0.74	0.113	4.85	5.28	0.43	0.283	0.31	0.540
50+	4.56	5.91	1.35	0.003	4.10	4.62	0.52	0.276	0.84	0.117
Sex										
Male	4.81	5.72	0.91	0.045	4.69	4.95	0.26	0.592	0.65	0.212
Female	4.64	5.94	1.30	0.000	4.37	4.98	0.62	0.046	0.68	0.116
Education										
Basic	4.00	5.17	1.17	0.019	4.71	4.07	–0.64	0.292	1.82	0.026
Intermediate	4.80	6.07	1.27	0.000	4.41	5.04	0.63	0.028	0.68	0.093
University	5.67	6.08	0.41	0.620	4.83	5.17	0.34	0.363	0.08	0.847
Health facilities										
Public	5.14	6.18	1.04	0.003	4.70	5.10	0.40	0.157	0.64	0.158
Private	4.15	5.52	1.37	0.005	4.88	4.41	–0.47	0.373	1.84	0.004
Drug stores	4.09	5.39	1.30	0.013	3.45	4.79	1.34	0.031	–0.04	0.827
Total	4.70	5.87	1.17	0.000	4.49	4.97	0.48	0.068	0.69	0.054

Notes: ^†^*p*-value of Wilcoxon’s test (adjusted with respect to intra-cluster correlation – ICC) comparing total knowledge scores between the characteristic groups before and after the interventions within the intervention/control groups.

^††^*p*-value of Mann–Whitney test (adjusted with respect to intra-cluster correlation – ICC) comparing total knowledge scores between the characteristic groups before and after the interventions between the intervention group and control group.

A nonparametric Mann–Whitney test for two related samples showed that the difference of the knowledge score improvements between the intervention and control groups was significant among HCPs with basic education and HCPs working in private health facilities.

Linear regression analysis using a backward stepwise model showed that the interventions and working in drug stores were the only independent factors that were associated with an improvement in knowledge scores, with coefficients equal to 0.696 (*p* = 0.005) and 0.663 (*p* = 0.038), respectively.

### Prescribing/dispensing patterns

Out of a targeted 2030 prescriptions, 2021 were collected (intervention arm: 1279; control arm: 742). The symptoms on prescribing/dispensing forms were categorized as (i) mild ARI: runny nose, cough and common cold, (ii) severe ARI: difficulty breathing or fast breathing and (iii) other diseases: all other symptoms. Table 4 shows that the improvement of antibiotic prescribing/dispensing practice was 28% for mild ARI in the intervention arm, compared with 3% in the control arm. All HCPs continued to have high rates of prescribing/selling antibiotics for severe ARIs. The results of the multilevel multiple regression model showed that the improvements of antibiotic prescribing/dispensing for mild ARIs were not significant – improvements were significant only for severe ARIs.

**Table 4.** HCPs’ prescribing/dispensing patterns before and after the interventions.

Prescriptions/dispensing (N)		Antibiotics used (%)	Multilevel logistic regression	
			I/C AOR (*p*-value) ^1^	A/B AOR (*p*-value)^2^
*Mild ARIs*				
Control (339)	Before (193)	140 (73)	1	1
	After (146)	102 (70)		0.310 (0.000)
	After – Before	(–3)		NA
Intervention (711)	Before (326)	275 (84)	1.003 (0.994)	1
	After (385)	215 (56)		0.310 (0.000)
	After – Before	(–28)		NA
*Severe ARIs*				
Control (115)	Before (49)	39 (80)	1	1
	After (66)	62 (94)		3.313 (0.101)
	After – Before	(14)		NA
Intervention (101)	Before (36)	36 (100)	30.113 (0.048)	1
	After (65)	64 (99)		3.313 (0.101)
	After – Before	(–1)		NA
*Other diseases*				
Control (288)	Before (143)	100 (70)	1	1
	After (145)	101 (70)		0.806 (0.235)
	After – Before	(0)		NA
Intervention (467)	Before (273)	205 (75)	1.204 (0.630)	1
	After (194)	133 (69)		0.806 (0.235)
	After – Before	(–6)		NA
*All diseases*				
Control (742)	Before	279 (73)	1	1
	After	265 (74)		0.556 (0.000)
	After – Before	(1)		NA
Intervention (1279)	Before	516 (81)	1.045 (0.876)	1
	After	412 (64)		0.556 (0.000)
	After – Before	(–17)		NA

Notes: ^1^I/C AOR: Adjusted odds ratio comparing intervention arm vs. control arm.

^2^A/B AOR: Adjusted odds ratio comparing after vs. before intervention acting. NA: non applicable.

## Discussion

Taken together, the three interventions significantly improved HCPs’ overall knowledge and practices regarding ARI and antibiotic use, and the improvement was significant compared with changes found in the control arm. Knowledge for ARI aetiology and severe ARI was improved after interventions, as were expertise and practical competence for cough and fever. In practice, prescriptions for severe ARIs remained high (99%), in line with IMCI recommendations. Antibiotic recommendations for mild ARIs decreased by nearly 30% in the intervention arm, but still remained on a comparatively high level (56%).

Previous educational interventions have proved effective for improving care, particularly when targeting HCPs through continued education, refresher training programmes and interactive case scenarios [4,25,26]. The most effective results have been seen when combining various interventions in a multi-faceted approach [27]. Delayed prescriptions have reduced antibiotic use by three quarters and have been successful in reducing ARI antibiotics, particularly for pick-up suggestions, ensuring the patient’s reciprocation [28]. The appropriate delay length, e.g. one week, and reinforcing or obstructing factors, e.g. patient expectations, could be investigated further.

The improvements regarding antibiotics for mild ARI were perhaps of a lower degree than anticipated. Knowledge and practical competence questions concerning cough and fever alone (i.e. mild ARI) increased significantly. However, after the interventions still only 44% of HCPs refrained from antibiotic use for mild ARI, indicating that other factors than knowledge seem more important in real practice. It is not surprising that it might be more complex and challenging to change real practice than to alter knowledge due to three reasons. Firstly, prescription patterns are influenced by many factors [29–31], creating an intricate cluster, where knowledge and practical competence are instances of numerous determinants of prescribing (another example could be financial incentives to prescribe). Secondly, changing prescribing behaviour is a process, which can be related to Prochaska’s ‘Stages of Change’ model [32], requiring more than simple gained understanding through a single intervention. Thirdly, the ‘Precede/Proceed’ model’s predisposing, enabling and reinforcing factors are vital for sustainable behaviour change, indicating that enhanced knowledge alone is insufficient to fully change prescribing behaviour [33].

Although these factors partially explain the low percentage (44%) of appropriate antibiotic use for mild ARIs in real practice, the results still indicate a need for additional context-appropriate multi-faceted interventions. Further qualitative research would be useful in providing more understanding of potential factors influencing the prescribing results seen in this study. In addition, a different intervention could be used to more effectively target the involvement of private health facilities, as their participation was lower in this study than the participation of public health facilities. Further qualitative work, as mentioned earlier, could help the development of an intervention specifically designed for private health facilities. It is vital to involve private health facilities in efforts to improve antibiotic use in Vietnam, as they form the majority of Vietnam’s drug dispensaries [18,34]. Point-of-care testing using e.g. C-reactive protein might reduce antibiotic use for non-severe ARIs in primary health-care in Vietnam [35].

This is one of few studies examining the effects of knowledge and practices regarding antibiotic use for children through a randomised controlled trial methodology. The study design attempted to control for the Hawthorne effect (i.e. improvement or modification in behaviour as a response of general attention, rather than resulting from the specific effect of the intervention) [21,33]. When studying the ARI intervention’s effect, the intervention in the control STI arm was assumed to induce a similar attention. The study therefore evaluated a ‘cleaner’ effect of the actual intervention content as the Hawthorne effect could be assumed to be relatively similar in both study arms. Another strength of this study is that the content of the interventions was evidence based, developed to meet the needs and requirements in the specific geographical area, for example focusing on misclassifications between mild and severe ARIs [14,18]. The interventions had been suggested in previous studies, including contextualized qualitative health behaviour research [14,18], which is crucial for developing successful interventions. A further strength is that actual prescription data was included as an outcome, as well as capturing self-reported practice in theoretical case scenario questions.

The generalizability of the study results must be considered carefully. In this case, the generalization of the results, the external validity, must be based on the theoretical propositions suggesting that the district is quite similar in relation to the phenomenon under study, e.g. antibiotic prescribing and dispensing for children with mild ARIs. Since there is little evidence to suggest that many rural areas within Vietnam are very different regarding antibiotic prescribing for children and dispensing practice than the area in the present study, the results can then be transferred to other similar contexts [36]. Differing participation rates indicate a need for a modified type of intervention to target the private sector. Self-completed questionnaires were relatively inexpensive, quick to complete, and permitted administrative flexibility; and confidentiality encouraged veracity, which strengthens the reliability of the study [37].

The long-term effects of the multi-faceted interventions were not evaluated due to the study’s budgetary limitations. However, it has been demonstrated in several studies that the intervention effect on prescribing behaviour can be maintained for years following the end of an intervention [38–40]. Nevertheless, it would not be unexpected if the HCPs slip back into old patterns of behaviour as many factors may influence the very high levels of antibiotic use for treatment of mild ARIs. In addition new health-care providers will also come to start work. Further research should focus on the structural sustainability of these interventions.

## Conclusion

The multi-faceted intervention significantly improved overall ARI knowledge and prescription patterns in the intervention group. Knowledge and practical competence for treatment of cough and fever improved after the interventions, as did knowledge for antibiotic-requiring symptoms and ARI aetiology, although improvements were less when put into real practice. We suggest a mixed method assessment and long-term follow up of these interventions in private versus public health facility settings to enable better appreciation of the effects and effect sizes of our interventions. Low-cost feasible interventions that are sustainable over a longer time period need to be developed and evaluated.

## Paper context

The problem of irrational antibiotic use has been reported in many countries, which have noted a rapid increase of antibiotic resistance in recent years. Misconceptions and subsequent practice of dispensing/prescribing antibiotics among health-care providers may be important factors behind the increasing antibiotic resistance. A multi-faceted intervention significantly improved overall ARI knowledge and antibiotic prescription patterns in the intervention group. Low-cost feasible interventions that are sustainable over a longer time period need to be developed and evaluated.
